# Hybrid Calcifying Epithelial Odontogenic Tumor and Ameloblastoma: A Report of an Extremely Rare Condition

**DOI:** 10.31661/gmj.v12i.3144

**Published:** 2023-12-17

**Authors:** Mohammad Saleh Ekrampoor, Hossein Daneste, Mohammad Amin Amiri

**Affiliations:** ^1^ Department of Oral and Maxillofacial Surgery, School of Dentistry, Shiraz University of Medical Sciences, Shiraz, Iran; ^2^ Student Research Committee, Shiraz University of Medical Sciences, Shiraz, Iran

**Keywords:** Ameloblastoma, Calcifying Epithelial Odontogenic Tumor, Odontogenic Tumors, Pindborg Tumor

## Abstract

Calcifying epithelial odontogenic tumor (CEOT) and ameloblastoma are types of odontogenic tumors accounting for 1%, and 10% of all the odontogenic tumors. While sharing same odontogenic origin, these tumors are found to exhibit distinct clinicopathological features. In the present study, we present the third hybrid CEOT/Ameloblastoma tumor ever reported. The current CEOT/Amelobastoma is occurred after a previously operated CEOT in the same area. The patient was refered with distict clinical features of swelling and paresthesia. In the radiographic examination, a unilocular lesion with mixed internal structure and ambiguous periphery was seen which exhibited buccal and lingual cortical expansion, thining, and perforation as well as inferior alveolar canal perforation. The histopathology results suggested a CEOT/Ameloblastoma lesion. After the tumor removal, the patient was set up for further follow-ups and maxillofacial prosthesis.

## Introduction

Calcifying epithelial odontogenic tumor (CEOT), also known as a Pindborg tumor, and ameloblastoma are among the well-known odontogenic tumors found in the maxillary-mandibular area. in this regard. CEOT is known as a locally aggressive and benign odontogenic tumor including 1% of all the odontogenic tumors [1][2]. This tumor is usually seen among adults with highest occurrence between the 3rd and 5th decade of life [3][4][5][6][7][8][9][10]. This tumor is most commonly seen in the premolar and molar region in the mandible with no gender predilection among the adult cases [1][10]. Half the cases are associated with an impacted tooth [10][11]. Clinically, CEOT can be found incidentally or may present as a slow-growing, painless swelling [1]. Radiologically, the destructive lesion appears radiolucent with variable calcification and can have a unilocular or multilocular cystic appearance [10][12]. These findings are not pathogonomic to CEOT and mimic ameloblastoma, dentigerous cyst, or other odontogenic tumors [13][14]. Concerning the clinical characteristics of ameloblastoma, it is found to be one of the most prevalent types of epithelial odontogenic tumors. This odontogenic tumor account for 1% of tumors and cysts in the jaws while constituting 10% of the odontogenic tumors [15][16]. Ameloblastoma originates from the dental lamina or enamel organ, stratified epithelium or epithelial remnants of the oral cavity, or epithelial lining of the odontogenic tumors [15][17][18]. This odontogenic tumor also exhibits no gener predilection with highest occurrence in mandibular angle and ascending ramus [15][19].

To the best of our knowledge, no evidence has ever pointed out to the possibility of CEOT and ameloblastoma having a same origin; nevertheless, two cases are reported to have hybrid CEOT/Ameloblastoma tumors [20][21]. In both of the cases the hybrid tumor was detected in the maxilla of Asian patients which were dignosed with histopathological examination. In the present study, we report the third case of the hybrid CEOT/Ameloblastoma tumor as a recurrence of a previously eradicated CEOT tumor.

## Case Presentation

**Figure-1:**
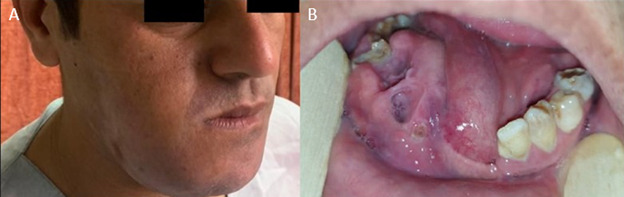
Figure 1. (A) Illustrates the extaoral view of the patient indicating a marked swelling on the right side of lower third part of his face. (B) Representation of an intraoral view of the patient suggesting the significantly enhanced volume of the right quadrant of the mandibular ridge resulting in less oral cavity space, and tongue displacement.

**Figure-2:**
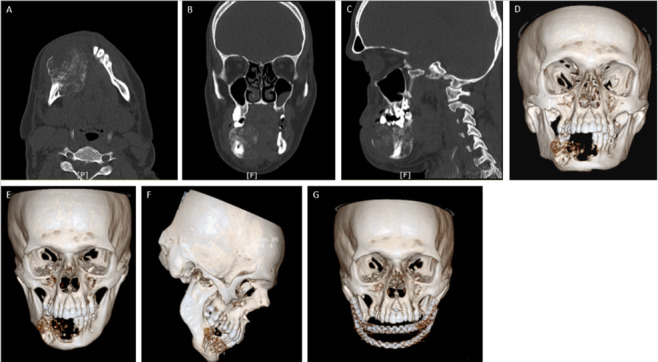
Figure 2. Representation of the lesion from the axial (A), coronal (B), and sagittal (C) view of CT scan. Also, the pre-operative 3D image of the lesion based on CT scan is illustrated in coronal (D, and E), and sagittal (F) views. Figure (G) illustrates the postoperative view of the patient after tumor removal.

**Figure-3:**
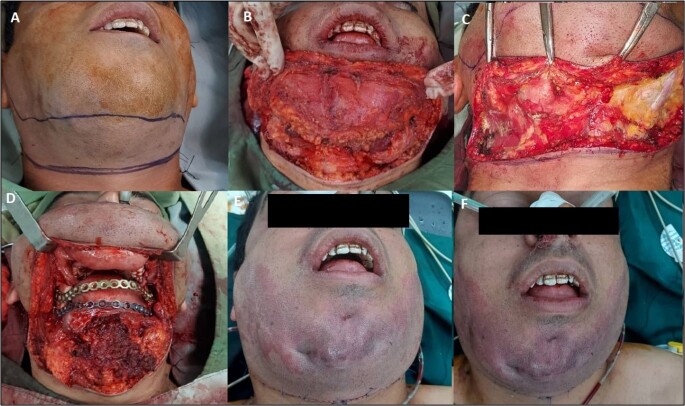
Figure 3. The pre- and post-operative views of the patient with marked areas of incision (A, E, F) as well as intraoperative views of the surgical area (B, C, D)

**Figure-4:**
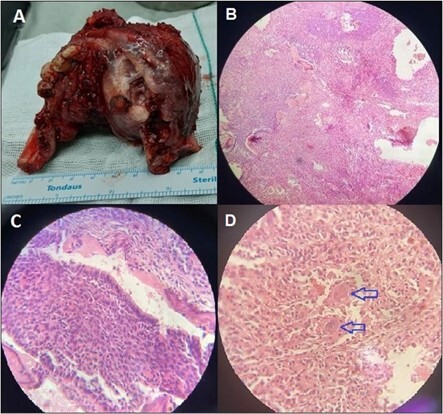
Figure 4. (A) Gross view of the resected area containing the CEOT/Ameloblastoma tumor. (B) Illustration of the histology section. A combination of epithelial cells containing the eosinophilic hyalinized areas with some calcifications in seen. (Magnification: 4X). (C) A compact area of angular cells with basophilic nuclei is seen. The reverse polarity of the cells surrounding the epithelial sheet is obvious. (D) A cross-section of the CEOT area is seen with two Leisgang ring calcifications as pointed out by the arrows.

A 32-year-old man was refered to the Oral and Maxillofacial Surgery Department of Rajaee Hospital in 2021 with significant swelling on the right side of the mandible (Figure-1). The patient had a history of a trauma in 2005, and over the next six years, the mandible’s right inner and outer mouth regions gradually began to swell. In 2011, the patient’s swelling was examined, and after removing seven teeth on the right side of the patient’s mandible, the lesion resulting from the swelling was totally resected, with the pathology result indicating CEOT. During the current episode, swelling of the right side of the mandible was seen, extending slightly beyond the midline. During the recent referral, the patient was reported to have psoriasis as his only underlying disease, with lesions on the face and both legs and scars caused by previous psoriasis lesions in the abdominal area. He had paresthesia and numbness on the right side of the mandible. More specifically, he reported numbness in the right area of the lower jaw and lower lip and less on the upper lip. All routine laboratory tests were normal.

Radiographic Interpretation

A computed tomography (CT) scan was requested (Figure-2), revealing a large expansile, destructive, predominantly lytic bone lesion in the anterior aspect of the right side of the mandible with limited extension to the contralateral side. Multiple septations were observed along with cortical expansion and erosion. No significant soft tissue component was detected. The mass extended inward into the oral cavity and projected into the face’s subcutaneous portion. Evidence of contrast enhancement was seen in the mass in favor of a vascular lesion. The possibility of intraosseous vascular malformation or hemangioma was considered. There were few prominent lymph nodes in the right submandibular area; the largest was about 17 x 10 mm. These findings indicated the diagnosis of CEOT.

Treatment Approach

Finally, the patient underwent surgery under general anesthesia in the supine position with blood pressure control (Figure-3). An apron incision was made in the mandibular region, and the myocutaneous flap was reflected. The pathologic lesion was exposed, explored, osteotomized, and excised. The sample was sent for pathologic evaluation. Then mandibular bone defect was reconstructed with two reconstruction plates. Hemovac® drains were inserted. After copious irrigation, the flap was repositioned, and the incision area was sutured in layers with Vicryl 3/0 and Nylon 5/0. A sterile dressing was applied. One intermaxillary fixation (IMF) screw was inserted in the mandible. The postoperative pathologic study revealed a final diagnosis of simultaneous CEOT and ameloblastoma. An apron incision was made in the mandibular region, and the myocutaneous flap was reflected. The pathologic lesion was exposed, explored, osteotomized, and excised. The sample was sent for pathologic evaluation. Then mandibular bone defect was reconstructed with two reconstruction plates. Hemovac® drains were inserted. After copious irrigation, the flap was repositioned, and the incision area was sutured in layers with Vicryl 3/0 and Nylon 5/0. A sterile dressing was applied. One intermaxillary fixation (IMF) screw was inserted in the mandible. The postoperative pathologic study revealed a final diagnosis of simultaneous CEOT and ameloblastoma.

Histopathology Results

The patient’s pathology results indicated a large 6 x 6 cm expansile mass that involved the mandible bilaterally and caused thinning and perforation of the cortices (Figure-4).

The cross-sections showed solid areas admixed with blood-filled cystic spaces. Microscopically, the tumor consisted of islands of odontogenic epithelium and showed predominantly follicular patterns of ameloblastoma. A single layer of palisaded columnar cells with reverse polarity surrounded the central core, which was composed of angular cells and cystic spaces. Other areas exhibited the basal cell pattern intermingled with the central core were sheets of large polyhedral epithelial cells with distinct borders and giant nuclei enclose areas of eosinophilic hyalinized material. Calcification was seen within this material. Large dilated vascular channels suggestive of vascular malformation were detected throughout the biopsy specimen. A final diagnosis of simultaneous calcifying epithelial odontogenic tumor and ameloblastoma was made. The patient provided consent for publishing this study, including patient images, on the condition of de-identification.

## Discussion

In this study, we have presented an extremely rare case of hybrid CEOT/Ameloblastoma recurred 10 years after eradication of a CEOT lesion in the same area. Based on the current literature, there are only two previously reported cases of CEOT/Ameloblastoma. The first case was a 53 year-old Asian man referring to the oral and maxillofacial surgery department in California [20]. The patient didn’t report any swelling or neural complications except erythematous gingiva during the intraoral examination [20]. The second case was a 62 year-old female patient in Turkey complaining of a mass on her left side of maxillary arch [21]. Now, hereby we report the third case with special characteristics in signs and symptoms. The patient was complaining of swelling in the right side of the anterior part of mandible with paresthesia which was possibly due to the extreme expansion of the CEOT/Ameoblastoma lesion towards the inferior alveolar nerve. Moreover, based on the radiographical examination, the patient had exhibited prominent lymph node in the ipsilateral side in the submandibular area. Moreover, it should be pointed out that all the three reported cases of CEOT/Ameloblastoma were Asian patients [20][21]. Moreover, the geographical distribution of ameloblastoma has indicated the same poattern regarding the higher incidence of ameloblastoma in the Asian and African population compared to the Caucasians’.

Since the patient had a history of mandibular surgery because of previous CEOT, it is possible to postulate that the current CEOT/Ameloblastoma lesion in the same area might be developed from the previous CEOT which has also differentiated into ameloblastoma; however, currently there is no solid evidence to confirm our hypothesis. The recurrence rate of CEOT has been reported 10% - 15% or 15% - 30% in the literature [3][22][23][24]. Moreover, until now, 7 cases of malignant CEOT or malignant transformation in CEOT is also reported in the literature [3][4][5][6][7][8][9]. Despite all this evidence, no study has ever suggested the possibility of CEOT cells trans-differentiating into ameloblastoma cells. However, our current case report may suggest a possible hypothesis in this regard.

Concerning the treatment approaches, it is stated that the common approach for CEOT is enucleation [25] and for ameloblastoma may vary from enucleation (with and without curettage) to radical resection [15][26]. The choice of the treatment option relies upon the level of the tumors aggressiveness [15]. Therefore, simple enucleation in some cases could result in possible recurrence of the lesion as well as higher chance of jaw fracture. This would be of high importance in the clinical setting since our case might have experienced an unusual recurrence leading to lower quality of life of the patient and planing a proper maxillofacial prosthesis to restore the facial structure.

## Conclusion

In the present study, we have presented a rare case of a 32 year-old man with CEOT/Amelobastoma tumor. The patient had indicated swelling and paresthesia during the clinical examination. During the radiographic examination, a multilocular lesion with mixed internal structure and ambiguous border resulting in cortical expansion, thining and perforation was seen. According to the clinical, radiographical, and histopathilogical results, the tumor was identified as CEOT/Ameloblastoma with agrressive pattern recurred 10 years after the first surgery for CEOT removal in the same area.

## Acknowledgement

The authors would like to express their gratitude to Dr. Shima Torabi for her kind advice and valuable support.

## Conflicts of Interest

The authors declare no conflict of interest.
